# Time course of adiponectin and its relationship to psychological aspects in patients with anorexia nervosa during inpatient treatment

**DOI:** 10.1371/journal.pone.0189500

**Published:** 2017-12-20

**Authors:** Magdalena Buckert, Esther Stroe-Kunold, Hans-Christoph Friederich, Daniela Wesche, Christiane Walter, Stefan Kopf, Joe J. Simon, Wolfgang Herzog, Beate Wild

**Affiliations:** 1 Department of General Internal Medicine and Psychosomatics, Medical University Hospital, Heidelberg, Germany; 2 Department of Psychosomatic Medicine and Psychotherapy, LVR-Clinics, University Düsseldorf, Düsseldorf, Germany; 3 Department of Endocrinology and Clinical Chemistry, Medical University Hospital, Heidelberg, Germany; Charité-Universitätsmedizin Berlin, Campus Benjamin Franklin, GERMANY

## Abstract

**Objective:**

The protein hormone adiponectin promotes metabolic and psychological health. The aim of the study was to track changes in adiponectin levels in response to weight gain and to assess associations between adiponectin and psychological aspects in patients with anorexia nervosa (AN).

**Methods:**

To investigate if adiponectin levels depend on AN severity, data were assessed from 11 inpatients with a very low body mass index (BMI) and a high chronicity (high severity group; HSS), and nine with less severe symptoms (LSS). During the course of treatment, serum adiponectin concentrations were assessed on a weekly basis along with BMI. Psychological variables (i.e., depression, anxiety, stress, and AN-specific symptoms) were obtained by means of electronic diaries. Longitudinal regressions and correlations were calculated to evaluate the temporal course of adiponectin and its relationship with psychological self-ratings.

**Results:**

At the beginning adiponectin was not increased in HSS patients (p = .56), and only marginally elevated in LSS patients (p = 0.07) compared with controls. In HSS patients, adiponectin increased along with BMI during the first treatment phase (i.e., when the BMI of patients was below 16 kg/m^2^) and thereafter decreased with further weight gain. In LSS patients, adiponectin was not associated with BMI increase. Furthermore, adiponectin was strongly negatively correlated with psychological self-ratings when the BMI of patients was above 16 kg/m^2^, i.e., higher levels of adiponectin were related to lower ratings of depression, anxiety, and AN-specific symptoms.

**Discussion:**

The study connects previous varying results by indicating that the course of adiponectin is dependent on BMI and symptom severity. Similarly, associations of adiponectin and psychological health depended on BMI.

## Introduction

Endocrine disturbances are prominent in anorexia nervosa (AN). Recently, there is growing interest in the role of peripheral hormones in AN. Specifically, adipose tissue has been recognized as an endocrine organ; peptides secreted by adipocytes, so-called adipokines, have been found to be altered in a range of mental disorders including eating disorders [1].

Unlike most adipokines, adiponectin′s concentration is inversely related to body fat [2]. Indeed, most studies that compared adiponectin levels in acutely ill anorectic patients as well as healthy controls found elevated levels in anorexia; however, reports of lower and comparable levels also exist (reviewed by [3]). The physiological function of high adiponectin levels in AN has not yet been established [4]. A causal role in the development of the disorder has been suggested [3]. However, others question the involvement of adiponectin in relation to weight loss in AN [5]. Aside from a potential role in the regulation of feeding behavior and energy homeostasis [6, 7], adiponectin also appears to be related to psychological functioning. Indeed, altered levels of adiponectin have been found in several other psychiatric conditions including in depression and anxiety disorders [1, 8]. In addition, there is preliminary evidence of a relationship between adiponectin levels and psychological factors, i.e., impulse control and social insecurity, in AN patients [9].

Studies on adiponectin changes during weight restoration are scarce and revealed conflicting results. Tagami et al. [10] observed an increase in adiponectin levels whereas Bosy-Westphal et al. [11] reported a non-significant decrease during weight gain. Two other studies assessed adiponectin levels on a more frequent basis. Both studies hint at nonlinear courses of adiponectin during weight recovery. Iwahashi et al. [12] took repeated measurements in a single case over a few months. They observed a gradual increase in adiponectin in association with weight gain until the BMI of the patient reached about 16 kg/m^2^. Thereafter, adiponectin levels decreased. A similar pattern was observed by Modan-Moses [13] in a sample of adolescent anorectic patients. Here, adiponectin was assessed at admission and again after one, three, and five months of hospitalization. Whereas during the first month adiponectin increased, five months after admission it decreased to a level below that measured at admission. In addition, Terra et al. [14] found that patients with a longer illness duration presented lower adiponectin levels. These various results give rise to the hypothesis that AN patients with a longer disease duration or higher symptom severity (and low BMI) present lower adiponectin levels than those with a lower symptom severity.

### Objectives of the present study

The aim of this study was (i) to assess serum adiponectin in AN patients with different levels of severity, (ii) to track changes in adiponectin levels in response to weight gain, and (iii) to investigate the longitudinal relationships between adiponectin and psychological variables in patients with AN during inpatient treatment. To this end, adiponectin was assessed weekly in 20 female anorectic inpatients. Psychological variables were assessed concomitantly using electronic diaries.

A main assumption of our approach was that one should differentiate between patients with a very low BMI and high symptom severity (HSS) and patients with a higher BMI and lower symptom severity (LSS). This distinction is important because recent studies have shown that in AN patients with HSS endocrine and emotional responses are different compared to patients with LSS [15, 16]. In addition, Terra et al. [14] reported that adiponectin levels depended on illness duration. Furthermore, Modan-Moses et al. [13] suggested that the heterogeneity of results regarding adiponectin changes during weight restoration could be explained by the severity of malnourishment at admission.

## Methods and materials

### Design

The study adopted a longitudinal design. Patients were informed about the study and written consent was obtained during the first week of inpatient stay. In total, 28 AN patients were enrolled (Note: A subgroup of these patients were subjects in previous investigations of the cortisol awakening response in AN patients [16] and in the time series of awakening cortisol [17]. Findings of this study regarding leptin have been previously published [18]). The study was approved by the medical ethics committee of the University Hospital Heidelberg (S404-2009). The authors assert that all procedures contributing to this work comply with the ethical standards of the relevant national and institutional committees on human experimentation and with the Helsinki Declaration of 1975, as revised in 2008.

At the beginning of the study, the Structured Clinical Interview for DSM-IV (SCID) was conducted. During the course of inpatient stay, serum adiponectin concentrations were assessed once per week while weight (BMI) was assessed twice per week (for the average number of measurements, please see Table 1). On the same day as adoponectin measurements were taken, patients answered questions on a handheld computer (at 1:00 p.m.) to assess a retrospective evaluation of the intensity of psychological symptoms during the course of the morning.

**Table 1:** Characteristics of the analyzed sample.

		HSS	LSS	Control Group
		n = 11	n = 9	n = 25
**age (years)**	***mean (SD)***	23.4 (3.8)	23.9 (4.0)	28.3 (5.5)
	***range***	(19–30)	(18–32)	(20.6–46.6)
**duration of illness (years)**	***mean (SD)***	5.1 (5.7)	3.7 (3.2)	—
	***range***	(1–17)	(1–10)	
**subtype AN-R**	***n (%)***	10 (90.9)	7 (77.8)	—
**BMI: first measurement (kg/m**^**2**^**)**	***mean (SD)***	13.6 (1.3)	16.2 (0.9)	21.2 (1.8)
	***range***	(11.8–15.7)	(14.4–17.5)	(18.0–25.0)
**BMI: last measurement (kg/m**^**2**^**)**	***mean (SD)***	17.0 (1.7)	17.1 (0.7)	—
	***range***	(14.5–19.0)	(16.0–18.4)	
**adiponectin: first measurement (μg/l)**	***mean (SD)***	11.3 (7.3)	13.4 (3.2)	10.1 (4.9)
	***range***	(3.7–23.8)	(9.2–19.8)	(3.6–23)
**adiponectin: last measurement (μg/l)**	***mean (SD)***	14.5 (4.8)	15.7 (4.7)	—
	***range***	(4.9–21.4)	(7.9–22.0)	
**duration of inpatient treatment (days)**	***mean (SD)***	128.9 (63.9)	68.2 (14.8)	—
	***range***	(52–231)	(42–84)	
**duration of participation (days)**	***mean (SD)***	114.8 (59.8)	64.8 (12.7)	—
	***range***	(39–223)	(39–83)	
**number of serum measurements**	***mean (SD)***	15.7 (8.4)	9.8 (1.9)	—
	***range***	(5–31)	(6–12)	
**mental health comorbidities**^a^ **(at enrolment)**	***n (%)***			
major depression (current)		4 (36.4)	6 (66.7)	0
minor depression (current)		1 (9.1)	1 (11.1)	0
panic disorder (current)		1 (9.1)	3 (33.3)	0
generalized anxiety (current)		1 (9.1)	0	0
social phobia (current)		2 (18.2)	0	0
obsessive compulsive (current)		0	1 (11.1)	0

HSS = patient group with high symptom severity; LSS = patient group with less severe symptoms; AN-R: restrictive subtype; SD = standard deviation; Out of N = 28 AN patients enrolled in the study, only N = 20 could finally be included in the data analysis (for reasons described in the text). Only the participants finally analyzed are described in this table.

^a^ diagnoses based on the Structured Clinical Interview for DSM-IV

### Participants

#### Patients

All participants were female AN inpatients meeting the DSM-IV criteria, over 18 years old with a BMI > 11 kg/m^2^ and sufficient physical and mental health to participate. Out of 28 recruited patients, the data of 20 patients could eventually be included in the longitudinal analysis. Out of the eight excluded patients, serum assessment for two of the patients was not possible for physical reasons; six patients dropped out of study participation. Subjects, who either passively or actively refused participation, did not differ significantly from those included in data analysis with respect to age (*p* = 0.344) or the first BMI measurement (*p* = 0.381; unpaired *t*-test, two-tailed).

The included 20 AN patients were admitted to two different wards. Eleven patients were recruited from an integrated psychosomatic and internal medicine ward (a group with high symptom severity, or HSS). This ward—specialized in the treatment of AN patients with a very low BMI and a longer illness duration—provides an intensive therapeutic schedule within a safe environment (e.g., supervised meal intake, weight management). The remaining nine patients were recruited from a psychotherapeutic ward (a group with less severe symptoms, or LSS) and were provided a multimodal psychodynamic-oriented treatment. Admission to the two different wards is determined by the severity and chronicity of the illness. Patients with a low BMI (usually lower than 15 kg/m^2^), medically endangered patients, and patients with either a chronic course of the disorder or frequent relapses were admitted to the HSS ward. The characteristics of the analyzed sample (N = 20) are described in Table 1.

#### Control group

Twenty-five healthy women served as the control group for the comparison of adiponectin levels with the AN patients. These participants were recruited via advertisements in the context of another study. They underwent the Structured Clinical Interview for the DSM-IV to ensure that those with lifetime diagnoses of mental disorders were excluded. All participants had a BMI between 18 kg/m^2^ and 25 kg/m^2^ and were aged 18 years or older. Also excluded were participants who had experienced significant weight fluctuations in the past six months or who were currently dieting. All participants provided written informed consent.

### Biochemical analyses

In AN patients, blood samples were taken in the course of the morning (between 10:00 and 12:00 a.m.) after a standardized breakfast between 7:30 and 8:30. Blood samples of the control group were taken at about 11:00 a.m. after a standardized breakfast at 9:30 a.m. Adiponectin was quantified in serum using a commercial immunological assay (Mediagnost, Reutlingen, Germany).

### Electronic diary measurements of psychological aspects

After enrolment, the patients received an electronic diary as well as training on how to use it. An alarm signal was used to remind the patients to complete the diaries. Questions assessing the degree of depression, anxiety, stress, pro-anorectic beliefs, and concern regarding eating were responded to. The items were adapted from psychometric questionnaires. Depression and anxiety were assessed by items taken from the Patient Health Questionnaire (PHQ-4; [19]). The intensity of pro-anorectic beliefs was measured by an item taken from the Pros and Cons of Anorexia Nervosa scale (P-CAN; [20]). Eating concern was measured using two items taken from the Eating Disorder Examination Questionnaire (EDE-Q; [21, 22]) assessing preoccupation with food and fear of losing control over eating. Concerning the assessment of stress patients were directly asked to rate their stress level in the course of the morning. Diary assessment was conducted as described in Stroe-Kunold et al. [23]. The diary items and details on item selection are provided in Table 2.

**Table 2:** Items assessing psychological aspects implemented in the electronic diary.

	item	scale
**depression**	“Over the last few hours, I have been feeling down, depressed, or hopeless”	PHQ-4
		subscale “depression” (r_it_ = 0.71)
**anxiety**	“Over the last hours I have been feeling nervous, anxious, or on edge.”	PHQ-4
		subscale “anxiety” (r_it_ = 0.63)
**stress**	“How would you rate your stress level this morning?”	^a^
**pro-anorectic beliefs**	“Over the last few hours, my anorexia has been making me feel secure.”	P-CAN
		subscale “safe/secured” (FL = 0.78)
**preoccoupation with food**	“Over the last few hours, thinking about food, eating or calories has been making it very difficult to concentrate on things I am interested in (for example: working, following a conversation, or reading).”	EDE-Q
		subscale “eating concern” (r_it_ = 0.77)
**fear of losing control**	“Over the last few hours, I have been experiencing a definite fear of losing control over eating	EDE-Q
		subscale “eating concern” (r_it_ = 0.77)

In representing the variable of interest (*r*_*it*_: item-to-total correlations; *FL*: factor loadings), items were chosen according to their psychometric properties. Additionally, we took into consideration which items would be clinically most adequate for daily assessment. *PHQ-4*: Patient Health Questionnaire-4 [19]. *P-CAN*: Pros and Cons of Anorexia Nervosa (P-CAN) scale [20]. *EDE-Q*: Eating Disorders Examination Questionnaire [21, 22].

^a^ Item not taken from a questionnaire.

### Data analysis

Data analysis was conducted using SAS 9.4^®^ (SAS Institute Inc., Cary, NC, USA). We used the PROC MIXED procedure to fit individual growth models (i.e., multilevel models) for adiponectin dependent on BMI over time. This allowed us to understand how adiponectin is associated with the change in BMI during inpatient treatment. By calculating unconditional linear growth models, we obtained estimates of the average slope (i.e., linear trend of adiponectin measurements dependent on BMI values) for the entire sample of participants (including a test of the null hypothesis that the slope is zero in the population); note: these models allow intercepts as well as slopes to vary across individuals [24]. We also estimated linear time trends for the change in BMI over time using PROC MIXED. Concerning the relationships between adiponectin and psychological processes, Pearson correlations were calculated. Note: for the correlation analysis BMI measurements were selected in accordance with time points of adiponectin assessment.

For HSS patients, analysis was conducted separately for the two phases of inpatient treatment. These phases were chosen according to the DSM-V specification of AN severity, i.e. during Phase I the severity of the patients was considered as “severe” or even “extreme” (BMI < 16 kg/m^2^), while during Phase II the severity of the patients ranged from “moderate” to “mild” (BMI ≥ 16 kg/m^2^). For LSS patients, data were analyzed for Phase II only (due to their BMI range).

## Results

### Comparison of adiponectin levels between patients and healthy controls

The comparison of the adiponectin measurements of patients at the beginning of the study with adiponectin measurements of the healthy control group revealed no significant difference between HSS patients and controls (t(34) = -0.59; p = .56), and showed only marginally elevated levels of adiponectin in LSS patients (t(32) = -1.86; p = .07). However, adiponectin of the patients at the end of the treatment was significantly higher in AN patients compared to the control group (HSS patients: t(33) = -2.39; p = .02; LSS patients: t(32) = -3.00; p = .005). BMI differed significantly between both patient groups and the controls at both measurement points (all p < .01). The respective descriptive statistics are provided in Table 1.

### Time course of adiponectin during inpatient treatment

**Fig 1:**
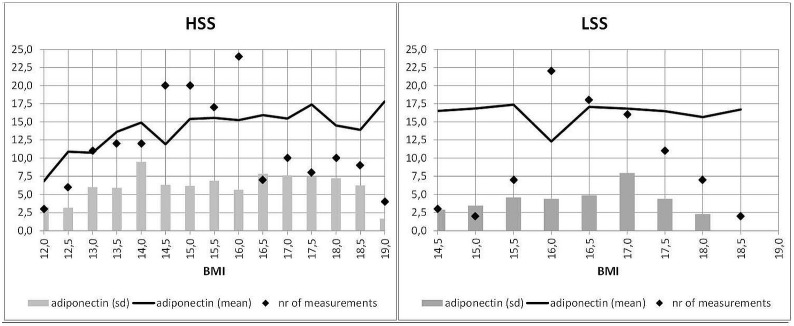
Average adiponectin serum concentrations (mean values) plotted against the BMI status of HSS and LSS patients (in increasing order). HSS = patient group with high symptom severity. LSS = patient group with less severe symptoms. Adiponectin was measured in μg/l. Standard deviations (sd) are plotted in grey, number of measurements are plotted as black dots. Note that statistical analyses in the LSS group were performed starting with BMI = 16 kg/m^2^.

Analysis of the slopes of adiponectin revealed evidence of nonlinear changes of this adipokine dependent on BMI (see Table 3, Model 1; Fig 1). Specifically, in HSS patients (BMI < 16 kg/m^2^) increasing BMI predicted an increasing trend in adiponectin during the first treatment phase (p = 0.06). After having reached a BMI ≥ 16 kg/m^2^, the association between adiponectin and BMI was reversed: adiponectin decreased with the increase of BMI. In LSS patients, no significant longitudinal association between adiponectin and BMI was obtained. BMI increased significantly with time in both treatment phases and in both patient groups, respectively (Table 3, Model 2). In line with the treatment foci of the two different wards, the increases in BMI among LSS patients were somewhat smaller than that among HSS patients.

**Table 3:** Linear time trends of longitudinal regressions during inpatient treatment.

	treatment phase	number of patients	Model 1		measurements per patient *mean (SD)*	Model 2		measurement per patient *mean (SD)*
			adiponectin = BMI			BMI = time		
			slope	p-value		slope	p-value	
HSS	I: BMI<16	11	**+1.80**	*0*.*06*	9.91 (7.41)	**+0.12**	**< .0001**	19.55 (15.10)
	II: BMI≥16	9	**-1.35**	**0.01**	7.11 (5.53)	**+0.10**	**< .0001**	13.44 (11.08)
LSS	II: BMI≥16	9	+2.65	0.16	8.00 (2.18)	**+0.05**	**0.0038**	16.44 (5.41)

HSS = patient group with high symptom severity; LSS = patient group with less severe symptoms. Treatment phases were chosen according to the DSM-V specification of AN severity (see details in text). Model 1 / Model 2 specify the longitudinal regression model. Significant slopes are printed in bold, trend-level significant slopes in italic.

### Relationship of adiponectin and psychological variables during inpatient treatment

Table 4 provides the partial correlation coefficients between adiponectin and the various psychological variables that were assessed concomitantly, controlling for BMI. Subjective ratings of actual depression, anxiety, and stress were highly significantly negatively associated with current adiponectin levels in HSS patients during the second treatment phase, i.e., BMI ≥ 16 kg/m^2^. That is, a higher self-assessment of depression, anxiety, or stress severity was associated with lower adiponectin levels. Similar but somewhat weaker associations were also found in LSS patients regarding depression and anxiety. During the first treatment phase in HSS patients, however, these associations were close to zero, and therefore not significant.

**Table 4:** Partial Pearson correlations (controlled for BMI) between adiponectin and psychological processes during inpatient treatment.

			Partial Pearson correlations (controlled for BMI) of adiponectin with the following factors											
sample	treatment phase	number of patients	depression		anxiety		stress		pro-anorectic beliefs		eating concern			
											preoccupation with food		fear of losing control	
			r	p-value	r	p-value	r	p-value	r	p-value	r	p-value	r	p-value
HSS	I: BMI<16	11	0.02	0.8235	0.03	0.7891	0.05	0.6247	**-0.22**	**0.0299**	-0.05	0.6495	-0.04	0.6581
	II: BMI≥16	9	**-0.45**	**0.0003**	**-0.71**	**< .0001**	**-0.84**	**< .0001**	**-0.38**	**0.0027**	**-0.62**	**< .0001**	**-0.44**	**0.0004**
LSS	II: BMI≥16	9	**-0.35**	**0.0046**	**-0.28**	**0.0245**	-0.18	0.1610	**-0.44**	**0.0003**	**-0.48**	**< .0001**	**-0.45**	**0.0002**

HSS = patient group with high symptom severity; LSS = patient group with less severe symptoms; r = Pearson’s correlation coefficient. Significant slopes are printed in bold, trend-level significant slopes in italic.

A similar pattern was obtained for anorexia-specific eating concerns. That is, adiponectin was significantly negatively associated with the EDE-Q items “preoccupation with food” and “fear of losing control” in both LSS and HSS patients during the second treatment phase, but not in the course of the first treatment phase. In contrast, significant negative associations of adiponectin and pro-anorectic beliefs were observed for both treatment phases in both patient groups.

## Discussion

For the first time, the current study has assessed adiponectin levels on a weekly basis in the course of treatment in two samples of anorectic inpatients differing in symptom severity. Furthermore, subjective ratings of depression, anxiety, stress, and anorexia-specific eating concerns were collected concomitantly. We found that time courses of adiponectin, as well as its association with psychological variables, depended on symptom severity and treatment phases as reflected in actual BMI levels.

Although the majority of cross-sectional comparison studies found elevated levels of adiponectin in anorectic patients, such a pattern was not obtained in our study. At the start of treatment, adiponectin levels were only marginally higher in LSS patients (with a mean BMI of 16.2 kg/m^2^ at the beginning); at this measurement point, there was no difference between HSS patients (mean BMI = 13.6 kg/m^2^) and controls. While this finding may seem contradictory at first glance, it fits quite well with previous studies when considering the actual BMI levels of the respective anorectic samples. In fact, the studies that investigated AN patients with a mean BMI between 13 and 14 kg/m^2^ (thus, being similar to our HSS patient group) also found no differences between patients and controls [9, 25], or even lower levels of adiponectin in patients [10]. In contrast, in the majority of studies reporting significantly elevated levels of adiponectin in AN patients, the mean BMI of patients was higher than 15 kg/m^2^, as was the case in our LSS patient group (see Khalil et al. [3] for an overview of comparison studies). Similarly, Terra et al. [14] reported a negative relationship between adiponectin levels and illness duration. Thus, whereas the negative relationship of adiponectin levels and BMI reported for the normal population [26] may span from overweight to underweight, it does not seem to extend to the severe malnutrition associated with HSS anorexia. A plausible reason for this could be the fact that adiponectin is produced mainly by adipocytes [27] that are markedly reduced in HSS patients. However, it is also possible that lower adiponectin levels in patients with a high symptom severity and a lower BMI might correspond to the physiologic response to severe starvation [10].

Regarding the course of adiponectin during treatment, the following pattern was observed: In severely ill (i.e., HSS) patients, adiponectin increased with increasing BMI during the first treatment phase and decreased thereafter (i.e., after the BMI of the patients reached 16 kg/m^2^). In LSS patients, no significant longitudinal relationship with BMI was obtained. The result in HSS patients is in line with both previous studies that assessed adiponectin at several time points during weight restoration. These studies also observed a decrease in adiponectin following initial increases after the first month of hospitalization [13], or when the BMI of the tracked patients reached a BMI of about 16 kg/m^2^ [12], respectively. Modan-Moses and colleagues [13] proposed that the initial increase in adiponectin in severely emaciated patients reflects early fat accumulation because young adipocytes display a higher rate of adiponectin gene expression compared to older ones. They furthermore assume that as soon as a critical threshold of body fat is reached, further increases in weight will be associated with a decrease in adiponectin analogous to the normal population. Thus, our results for HSS patients support the model proposed by Modan-Moses et al. [13] based on a reliable data set with multiple measurements for each patient. However, there was no decrease of adiponectin dependent on increasing BMI in LSS patients as would have been predicted by this model. This finding is in line with a recent study that reported no difference in adiponectin levels between treatment naïve AN patients (mean BMI 15.6 ± 1.1 kg/m^2^) and a group of re-fed AN patients [28]. A possible explanation may be given by the fact that the increase in BMI was considerably lower in LSS patients compared to HSS patients. It could also be possible that the assumed optimal fat mass for adiponectin secretion was not reached [14]. However, it could also suggest that—modulated by illness severity—other factors may also influence adiponectin secretion [13].

It should be noted that despite the significant decrease of adiponectin during the second treatment phase, the mean adiponectin levels of anorectic patients at discharge were significantly higher than those of controls. This may simply be explained by the fact that mean BMI was still significantly lower in patients at this measurement point. In summary, BMI indeed seems to be an important moderator of adiponectin changes during treatment. These factors should also be considered when comparing mean adiponectin levels of AN patients to controls.

An interesting finding of the current study is the high and consistent relationship of adiponectin levels with psychological factors, particularly depression and anxiety, in AN patients with a BMI ≥ 16 kg/m^2^. Low levels of adiponectin have been found in several psychiatric conditions, including in severe depression [8, 29], anxiety disorders [30], and bipolar disorder [31]. Furthermore, animal studies have provided experimental evidence for an antidepressant effect of adiponectin [32]. Thus, the high negative correlation between adiponectin levels and the concomitant subjective ratings of depression and anxiety are well in line with these observations. Several potential mechanisms of adiponectin′s central anti-depressant and anxiolytic actions have been proposed. For example, adiponectin receptors are expressed in the limbic system [32, 33], and adiponectin has been found to be related to hippocampal neurogenesis [34]. Another potential mechanism regarding how adiponectin exerts anti-depressant effects may be the inhibition of pro-inflammatory cytocines [35]. Furthermore, reduced levels of adiponectin seem to impair glucocorticoid-mediated negative feedback regulation of the hypothalamus-pituitary-adrenal axis [32]. In addition, there is evidence that chronic stress and glucocorticoids inhibit adiponectin gene expression [32, 36, 37]. The latter mechanism may explain the very high negative correlation between adiponectin and the subjective ratings of stress in HSS patients during the second treatment phase. However, there is no obvious explanation for why the correlation of adiponectin and stress, despite being the highest in HSS patients (BMI ≥ 16 kg/m^2^), was not significant in LSS patients while the correlations with anxiety and depression were significant (albeit slightly smaller) in this group as well. Of note, none of these correlations was evident in HSS patients during the first treatment phase, i.e., when their BMI was below 16 kg/m^2^. Thus, one may speculate that a certain weight threshold needs to be reached before adiponectin can exert an influence on these psychological factors [cf. also 12]. An alternative explanation may be the impaired ability of severely ill AN patients to recognize their emotional state [38, 39].

Interestingly, similar negative correlations were also observed for anorexia-specific eating concerns. That is, the lower the current adiponectin level, the higher the fear of losing control, the preoccupation with food, and pro-anorectic beliefs. Of note, the latter correlation was the only one that was also significant in the first treatment phase of HSS patients—most likely because these beliefs are accessible to patients even in a state of emotional numbing. Thus, analogously to depression and anxiety disorders, adiponectin may also be related to beneficial psychological functioning in the context of anorexia nervosa.

Taken together, our study contributes to recent research focusing on the role of peripheral peptides in eating disorders [40–46]. For example, the gastric hormone ghrelin was found to be associated with physical activity in AN [43]. NUCB2/nesfatin-1, an anorexigenic hormone that peripherally occurs in adipose tissue, was reported to be correlated with anxiety among anorectic [40] as well as—in a sex-dependent manner—obese patients [42]. However, contrary to our findings regarding adiponectin, NUCB2/nesfatin-1 was not associated with depressiveness or perceived stress [40]. This might suggest a broader involvement of adiponectin in psychological well-being in the context of AN.

### Limitations

In this study, we report results regarding serum levels of whole adiponectin. However, several isoforms of circulating adiponectin exist that may exert differential functions [10, 14, 32]. Thus, it would be of great value to distinguish those specific isoforms (as was already done in [9, 13, 14, 28]) in future longitudinal studies. Future studies may also benefit from including repeated measures of appetite, e.g., by means of visual analogue scales.

Furthermore, body fat was not measured in our study and therefore, we could only track changes of adiponectin dependent on BMI and not on fat mass. In addition, our sample size could be considered rather small. On the other hand, the number of measurement points was very high, thereby enabling the close tracking of individual adiponectin levels.

Much more research is needed to elucidate the mechanism linking adiponectin and psychological factors observed in this study. For example, it is still a matter of debate whether or not adiponectin enters the brain from the periphery, and what effects it exerts in the CNS on a cellular level [33].

## Conclusion

This study has, for the first time, assessed adiponectin levels on a weekly basis in two groups of AN patients during their inpatient stay. In HSS patients, adiponectin increased during the first treatment phase (i.e., until the BMI reached 16 kg/m^2^) and decreased thereafter, while no such association was observed for LSS patients. Interestingly, when the BMI was 16 kg/m^2^ or higher, self-ratings of depression, anxiety, stress, and AN-specific concerns were strongly negatively associated with adiponectin levels in both patient groups. This finding raises the interesting question whether or not adiponectin may exert positive influences on the mental state of AN patients.

## Supporting information

S1 File Repeated measurement data including adiponectin, BMI, and psychosocial variables. (XLSX)

S2 File Adiponectin data of both the patient and the control group. (XLSX)
